# Transient bone marrow hypoplasia preceding T-Cell acute lymphoblastic leukemia: a case report

**DOI:** 10.4314/ahs.v21i2.25

**Published:** 2021-06

**Authors:** Ernest Naturinda, Paul George, Joseph Ssenyondwa, Deogratias Bakulumpagi, Joseph Lubega, Peter Wasswa

**Affiliations:** 1 Baylor College of Medicine Children's Foundation Uganda, Kampala, Uganda; 2 Baylor College of Medicine and Texas Children's Hospital, Houston, TX. USA

**Keywords:** Pancytopenia, hypoplasia, aplastic anemia, T-cell acute lymphoblastic leukemia, case report

## Abstract

**Background:**

Acute lymphoblastic leukemia (ALL) is the most common childhood malignancy and is characterised by hyperproliferation of malignant lymphocytes in the bone marrow. Rarely, ALL may be preceded by a period of pancytopenia and bone marrow hypoplasia which spontaneously recovers. This phenomenon, which has not before been described in T-cell ALL, is referred to as transient bone marrow hypoplasia.

**Case presentation:**

A 5-year-old boy who presented with high-grade fever and generalised lymphadenopathy, was found to have pancytopenia on peripheral blood count and bone marrow hypoplasia. He was observed over a one-month period during which his bone marrow and peripheral blood counts recovered spontaneously. Symptoms recurred after 4 months and he was found to have blast infiltration of the bone marrow and diagnosed with T-cell ALL.

**Conclusion:**

Cases of transient bone marrow hypoplasia or overt aplastic anemia with spontaneous recovery and then followed by B-cell ALL or Acute Myeloid Leukemia have been described previously in the medical literature. This is the first case of transient bone marrow hypoplasia resulting into ALL of T-cell immunophenotype. While marrow hypoplasia preceding ALL remains poorly understood, it suggests an antecedent environmental insult to lymphoid progenitors or a germline abnormality that predisposes to lymphoid dysplasia. This may provide clues to the hitherto unknown pathophysiological process and etiological factors that precede the majority of childhood ALL cases. This case enlightens pediatricians about the existence of such rare cases so as to periodically follow up children with pancytopenia and/or bone marrow hypoplasia for prolonged periods even after apparent recovery.

## Introduction

Childhood acute lymphoblastic leukemia (ALL) is the most common pediatric cancer worldwide^1^. It is characterized by an abnormal proliferation of arrested immature lymphoid precursors (lymphoblasts), resulting in anemia, thrombocytopenia, and/or neutropenia^2^. Clinically, the typical presentation of ALL consists of fever, lymphadenopathy, bruising, bleedingor bone pain, with rapid progression and often without an apparent prodrome^3^. In rare cases, however, a transient prodrome of hypoplasia or aplasia precedes overt leukemia^4^.

Whereas the bone marrow of children with acute leukemia is characterized by proliferation of malignant blasts, patients who undergo the phenomenon of transient bone marrow hypoplasia preceding leukemia initially present with clinical features of pancytopenia including on peripheral blood examination. The bone marrow is hypoplastic or aplastic and, therefore, they are often initially diagnosed with bone marrow failure^5^. The majority of cases of transient marrow hypoplasia preceding leukemia occur in females and in children less than 10 years of age^6^. Fever and other signs suggestive of infection are commonly reported prior to the onset of the aplastic phase. In most patients, the marrow hypoplasia is transient, with spontaneous or steroid-induced recovery of the bone marrow and peripheral counts. However, typically within a six-month period, these patients present with features consistent with acute leukemia, and their peripheral blood and/or bone marrow shows a proliferation of blasts, confirming a diagnosis of acute leukemia^7^. Despite these differences in presentation, the hypoplastic phase preceding ALL does not seem to confer a worse prognosis and does not change the established standard of care for leukemia in general^8^.

Relatively few cases of this phenomenon have been described worldwide, and none to our knowledge in African children. Further, transient marrow hypoplasia preceding leukemia has been previously described as occurring with B-cell ALL and Acute Myeloid Leukemia. We found no cases of transient marrow hypoplasia preceding T-cell ALL in published literature. Here, we present a case of pediatric T-cell ALL that was preceded by a period of bone marrow hypoplasia in a 5-year-old boy.

## Case

A 5-year-old, previously healthy boy presented to the Pediatrics Emergency Room with a six months history of high-grade fevers, oral sores and generalized lymphadenopathy. Other symptoms included night sweats and weight loss, with no known history of bruising or bleeding. He had been previously presumptively treated for Tuberculosis without any significant improvement. There was no known history of steroid use. At initial presentation, he was ill-appearing, pale and had generalized lymphadenopathy. A complete blood count showed a white cell count of 1,750 cells/µL, neutropenia of 510 cells/µL, lymphopenia of 770 cells/µL, and monocytosis of 470 cells/µL. He had thrombocytopenia of 21,000 platelets/µL and anemia with hemoglobin concentration 7.0 g/dL. The initial differential diagnosis included aplastic anemia and leukemia. A bone marrow aspirate and biopsy were performed that showed a hypocellular marrow with reduced erythroid progenitors, relative lymphocytosis with aberrant morphology of small mature lymphocytes but no blasts. Flow cytometry revealed no abnormal clonal proliferation. Conclusively, findings were suggestive of a hypoplastic marrow of undetermined etiology.

Although we considered performing a work up for virus infections and genetic bone marrow failure syndromes, our center does not have capacity for these studies. He was managed with antibiotics in view of fever and moderate neutropenia and supportive packed erythrocyte transfusions. Over the subsequent three weeks, his symptoms resolved, his blood counts recovered to within normal ranges, and he was given a follow up appointment in one month. Noteworthy, he was not treated with steroids during this period.

The patient is reported to have been generally well following discharge until 4 months later when he had a recrudescence of high-grade fevers, pallor, and generalized lymphadenopathy. His complete blood count showed leukopenia with white blood cells 530/µL, profound neutropenia 60/µL, lymphopenia 430/µL, monocytes 40/µL, normal platelet count of 196,000/µL, and moderate anemia with hemoglobin 9.5 mg/dL. Bone marrow aspirate showed 80% lymphoblasts. Flow cytometry of bone marrow aspirate showed clonal proliferation of cells positive for CD45, CD3, CD7, CD5 and CD2. The cells were negative for B cell markers CD19, C20, and CD10 and negative for myeloid markers CD13 and CD33, consistent with T-cell ALL (Figure 1). Analysis of cerebrospinal fluid was negative for pleocytosis or blasts. He was risk-stratified as High Risk ALL based on T cell immunophenotype and started on a 4-drug induction of vincristine, dexamethasone, daunorubicin, L-asparaginase and intrathecal methotrexate. He achieved remission with no evidence of minimal residual disease at a flow cytometry resolution of 1 in 10,000 events. He successfully completed augmented BFM Consolidation, Capizzi methotrexate in Interim Maintenance, and Delayed Intensification, and is on Maintenance chemotherapy as per Children's Oncology Group treatment protocols. He was clinically in good general condition with no evidence of relapse at last review.

## Discussion

ALL preceding spontaneous recovery of unexplained bone marrow hypoplasia is a rare and highly hypothesized phenomenon^9^,^10^. It occurs in approximately 2% of cases of ALL, usually amongst females below 10 years of age^9^–^12^. This is the first and only case observed at our pediatric cancer center over the last three years. Several terms have been used by several authors to describe this phenomenon, including pancytopenic prodrome of ALL, hypoplastic anemia, transient pancytopenia preceding ALL (pre-ALL), or aplastic anemia preceding ALL)^11^–^16^. Some literature, however, describes findings suggestive of aplastic anemia in pre-ALL as “nonclassical” or inadequate to suggest aplastic anemia^10^,^11^. Typically, no other known bone marrow failure syndromes such as Fanconi anemia or myelodysplasia can be identified using morphological, cytogenetic or immunophenotypic analyses. In some cases, an inconsequential percentage of blasts may be found^11^,^16^. In most cases, medical history is significant for a fever preceding marrow hypoplasia and an infection-related cause can be identified in some cases^9^,^10^,^14^. The period from marrow recovery to overt ALL is typically within 6 months, consistent with our patient who developed overt ALL within 4 months^9^,^14^.

The pathophysiology and biological underpinnings of the pre-ALL remain enigmatic^11^. Hypotheses include virus-induced bone marrow suppression and concomitant leukemogenic genetic damage, or an aberrant immune response to virus infection that perhaps enhances proliferation of pre-leukemia lymphoblasts to overt leukemia^16^,^18^ – ^20^. Conversely, literature has described cases of ALL remissions associated with infection suggesting that pre-ALL may be a case of transient remission of undiagnosed ALL. This is the first case of pre-ALL of T cell immunophenotype. The reason for the predominance of B cell immunophenotype among cases of pre-ALL is not apparent but it may relate to the rarity of T cell ALL (<20% of ALL) and/or the extramedullary origin of T lymphocytes^22^.

Bone marrow hypoplasia preceding ALL and its unresolved mechanisms warrant further research because it may open new insights into etiology of pediatric ALL and enable pre-overt detection of ALL that may be more curable on the basis of a lower leukemia cell burden in the marrow during this period^16^. This case also enlightens practitioners to periodically follow up children with unexplained pancytopenia and/or bone marrow hypoplasia for several years even after apparent recovery.

## Limitations

We are currently unable to do genetic and viral studies at our facility. Availability of such workup would have gone a long way in studying underlying genetic mutations, probable oncogenic viruses associated with this condition.

## Declaration

### Availability of data and materials

Available.
